# Genomic analysis of mutations in platelet mitochondria in a case of benzene-induced leukaemia: A case report

**DOI:** 10.1097/MD.0000000000024014

**Published:** 2021-01-08

**Authors:** Dianpeng Wang, Xiangli Yang, Diya Cai, Peimao Li, Zhimin Zhang, Dafeng Lin, Yanfang Zhang

**Affiliations:** a Shenzhen Prevention and Treatment Center for Occupational Diseases, Shenzhen, Guangdong; b Hebei North University, Hebei, China.

**Keywords:** benzene, genome, leukaemia, mitochondrion, platelet

## Abstract

**Introduction:**

As a hematopoietic carcinogen, benzene induces human leukemia through its active metabolites such as benzoquinone, which may cause oxidative damage to cancer-related nuclear genes by increasing reactive oxygen species (ROS). Mitochondrion is the main regulatory organelle of ROS, genetic abnormality of mitochondrion can impede its regulation of ROS, leading to more severe oxidative damage. Mutations have been related to certain types of cancer in several mitochondrial genes, but they have never been completely analyzed genome-wide in leukemia.

**Patient concerns:**

The patient was a 52-year-old female who had chronic exposure to benzene for several years. Her symptoms mainly included recurrent dizziness, fatigue, and they had lasted for nearly 8 years and exacerbated in recent weeks before diagnosis.

**Diagnosis:**

Samples of peripheral blood were taken from the patient using evacuated tubes with EDTA anticoagulant on the second day of her hospitalization. At the same time blood routine and BCR/ABL genes of leukemic phenotype were tested. Platelets were isolated for mitochondrial DNA (mtDNA) extraction. The genetic analysis of ATP synthase Fo subunit 8 (complex V), ATP synthase Fo subunit 6 (complex V), cytochrome c oxidase subunit 1 (complex IV), cytochrome c oxidase subunit 2 (complex IV), cytochrome c oxidase subunit 3, Cytb, NADH dehydrogenase subunit 1 (complex I) (ND) 1, ND2, ND3, ND4, ND5, ND6, 12S-RNA, 16S-RNA, tRNA-Cysteine, A, N, tRNA-Leucine, E, displacement loop in platelet mtDNA were performed. All the detected gene mutations were validated using the conventional Sanger sequencing method.

**Interventions:**

The patient received imatinib, a small molecule kinase inhibitor, and symptomatic treatments.

**Outcomes:**

After 3 months treatment her blood routine test indicators were restored to normal.

**Conclusion:**

A total of 98 mutations were found, and 25 mutations were frame shift. The ND6 gene mutation rate was the highest among all mutation points. Frame shifts were identified in benzene-induced leukemia for the first time. Many mutations in the platelet mitochondrial genome were identified and considered to be potentially pathogenic in the female patient with benzene-induced leukemia. The mutation rate of platelet mitochondrial genome in the benzene-induced leukemia patient is relatively high, and the complete genome analysis is helpful to fully comprehend the disease characteristics.

## 1 Introduction

Benzene can cause leukemia, therefore is internationally recognized as a carcinogen. ^[1,2]^ When it is metabolized into ultimate carcinogenic toxicants by enzymes like cytochrome P450,^[3]^ the redox reaction concomitantly occurs to produce superoxide anion radicals, hydrogen peroxide, hydroxyl radicals and other free radicals collectively referred to as reactive oxygen species (ROS).^[4]^ Increased ROS can cause oxidative stress which is hazardous to the biological system and can mediate key events in the development of leukemia stem cells including genetic induction of key oncogenes, hematopoietic stem cell chromosomal abnormalities, genomic instability, stromal cell disorders, hematopoietic stem cell and interstitial cell apoptosis.^[4]^ These key events combined with other carcinogenic mechanisms may eventually lead to the occurrence of leukemia.

In fact, the predominant target of ROS in cells is the mitochondrion.^[5]^ Mitochondria as the primary source of energy production are very important organelles in most eukaryotic cells. They carry their own set of genetic instructions outside the nuclei, mitochondrial DNA (mtDNA), to perform aerobic respiration. The mtDNA, which form a closed double-stranded circular structure, comprise 37 genes in the length of 16569 bp, encoding 13 polypeptides that form 4 complexes involved in the oxidative phosphorylation, 22 tRNA necessary for protein synthesis in mitochondria, and 2 rRNA.^[6]^ The mtDNA lack protection of histone and DNA repair system. When they are exposed to high-level ROS produced by the respiratory chain or metabolism of xenobiotics such as benzene, damage will happen much easier to the mtDNA than to the nuclear DNA. It is estimated that the mutation frequency of the mtDNA is over ten times higher than that of the nuclear DNA. Mutations in mtDNA are the main reason for the abnormality of mitochondria.^[7]^

Unlike the nuclear genome, the mitochondrial genome has no intron and is only composed of encoding genes and regulatory sequences, thus one point mutation in the mtDNA may cause alteration of important structural genes, leading to changes in the expression or property of the expressed proteins.^[8]^ When abnormality happens in the mtDNA encoded proteins that are involved in the electron transport chain, mitochondrial free radicals will not be effectively removed, and their accumulation will further trigger oxidative stress in mitochondria and the whole cell.^[9]^

The role of mtDNA mutation in disease development has attracted the attention of scholars worldwide. At present, more and more studies have found that mitochondrial gene mutations and gene expression abnormalities can cause a variety of diseases such as cancer and diabetes.^[10]^ Single point mutations in mitochondrial genes were also linked to the risk of leukemia in various experimental studies,^[11]^ however, they have never been completely analyzed genome-wide in leukemia patients.

Mitochondrion is one of the major organelles in the platelet which is free of somatic nuclei. The DNA extracted from pre-isolated platelets is mostly mtDNA and much less interfered by the homologous sequences of nuclear DNA during the analysis. Thus, platelet-extracted DNA is an ideal material for analyzing mitochondrial genome.^[12]^ This case study was intended to analyze the characteristics of mutations in platelet mitochondrial genome in a patient suffering from benzene-induced leukemia.

## 2 Case presentation

A 52-year-old female was diagnosed as chronic myelogenous leukemia (chronic phase) based on peripheral blood and bone marrow examination according to Guidelines for diagnosis and treatment of chronic myeloid leukemia (CML) (Diagnosis of occupational tumor GBZ 94–2017, 2017 Edition and Diagnosis of occupational benzene poisoning GBZ 68–2013, 2013 Edition) issued by National Health Commission of the People's Republic of China (http://www.nhc.gov.cn/wjw/pyl/201706/0219719a46fb469e875837f18d64b109.shtml). When she was hospitalized in July 2017, her peripheral blood analyzing results showed that the white blood cell count was 50.38 × 10^9^/L (reference value: 3.5 - 9.5  × 10^9^/L), the neutrophil count was 29.93 × 10^9^/L (reference value: 1.8 - 6.3  × 10^9^/L), the hemoglobin concentration was 80.8 g/L (reference value: 115 - 150 g/L), and the platelet count was 961 × 10^9^/L (reference value: 125 - 350  × 10^9^/L). Her bone marrow biopsy at the same time showed that the number of medium and late granules and rod-shaped granulocytes were increased and the percentage of primary cells (type I + type II) was 4% (reference value: < 0%). These parameters are necessary for disease diagnosis and classification.

Her medical history recorded that she did not have hepatitis, tuberculosis infection, surgery, trauma, blood transfusion, and drugs and food allergy. She had regular menstrual cycles since 19 years old. She had worked in benzene-exposed factories for more than 10 years. Family history recorded that her relatives had no infectious diseases and genetic diseases (including leukemia and other cancers).

### 2.1 Diagnostic and genetic assessment

BCR/ABL fusion gene is a mutation that is formed by the combination of 2 genes, known as BCR and ABL. BCR/ABL fusion gene test is most commonly used to diagnose or rule out CML. Fluorescence in situ hybridization of her bone marrow showed that the BCR/ABL fusion gene was positive. Platelets were isolated from 2 mL EDTA anticoagulant venous blood by centrifugal extraction method (100 × g, 10 min). MtDNA was then extracted from the isolated platelets with Ezup column blood genomic DNA extraction reagent (China Biotechnology Engineering Company, Shanghai) according to the manufacturer's protocol. Polymerase chain reaction (PCR) primers were designed for mitochondria. Gene amplification was performed in an Applied Biosystems steponeplus PCR instrument. PCR was performed in a 40 μL volume containing 20 μL of 2 × PrimeSTAR HS (Premix) (cat#R040A, Takara, China), 1 μL of each primer (10 pmol; Sangon Biotechnology, China) (Supplementary Table 1), 14 μL of H_2_O and 4 μL MtDNA.

A 3-step PCR was performed. The initial denaturation at 95°C for 1 minute was followed by 15 cycles of denaturation at 95°C for 10 second, annealing at 58°C for 30 second, extension at 72°C for 120 second, and 25 cycles of denaturation at 95°C for 10 s, annealing at 60°C for 30 second, extension at 72°C for 120 second. Each amplified fragment was certified and purified using an Agarose Gel DNA Fragment Recovery Kit (Sangon Biotechnology, China) (Supplementary figure 1), and subsequently sequenced using an ABI PRISM 3730 sequence analyzer (Sangon Biotechnology, China). The obtained sequences were aligned with a multiple sequences alignment interface CLUSTAL-X to compare with standard mitochondrial sequence (NC_012920.1). Identified mutations were confirmed by repeated analysis of both standard mitochondrial sequence and mitochondrial database (MITOMAP).

### 2.2 Treatment and outcomes

The patient received imatinib, a small molecule kinase inhibitor, and symptomatic treatments. After 3 months of treatment her blood routine test indicators were restored to normal. The peripheral blood analyzing results showed that the white blood cell count was 3.82 × 10^9^/L, the neutrophil count was 2.35 × 10^9^/L, the hemoglobin concentration was 125.0 g/L, and the platelet count was 146 × 10^9^/L. Her bone marrow biopsy showed no abnormalities. BCR/ABL fusion gene from her bone was negative. The platelet mitochondrial complete genome NCBI-BLAST search analysis efficiency was 99.8%. The mutation rate from high to low was that of NADH dehydrogenase subunit 1 (complex I) (ND)6, Cytb, displacement loop (D-Loop), ND5, A, cytochrome c oxidase subunit 1 (complex IV), ND3, ND4, ribosomal RNA small subunit, ribosomal RNA large subunit, tRNA-Cysteine, N, cytochrome c oxidase subunit 2 (complex IV), ATP synthase Fo subunit 6 (complex V), cytochrome c oxidase subunit 3, ND1, ND2, tRNA-Leucine, ATP synthase Fo subunit 8 (complex V). Analysis of the sequence data showed a total of 98 mutations including 58 point mutations, 6 insertions, 9 deletions, 25 frame shift mutations, 24 synonymous, 22 noncodings and 16 missenses. The ND6 gene mutation rate was the highest among all detected genes. The detailed results were shown in Table 1. ATP synthase Fo subunit 8 (complex V), gene included 1 synonymous. ATP synthase Fo subunit 6 (complex V) geneincluded 2 missenses. Cytochrome c oxidase subunit 1 (complex IV) gene included 3 synonymouses. Cytochrome c oxidase subunit 2 (complex IV) gene included 1 missense and 1 synonymous. Cytochrome c oxidase subunit 3 gene included 2 synonymous. Cytb gene included 9 frame shift mutations, 2 missenses and 3 synonymouses. NDI gene included 1 synonymous. ND2 gene included 1 synonymous. ND3 included 2 missenses and 1 synonymous. ND4 gene included 3 synonymous. ND5 gene included 1 deletion, 1 frame shift mutation, 1 synonymous and 1 missense. ND6 gene included 15 frame shift mutations , 2 noncodings, 7 synonymouses and 8 missenses. Ribosomal RNA small subunit gene included 2 point mutations. Ribosomal RNA large subunit gene included 1 deletion mutation. tRNA-Cysteine gene included 2 noncodings. A gene had 1 insertion and 2 deletions. N gene had 1-point mutation and 1 deletion. tRNA-Leucine gene included 1 noncoding. E gene had 1-point mutation. D-Loop had 11 point mutations and 1 deletion and 5 insertions and 17 noncodings.

**Table 1:** Platelet mitochondrial gene mutation statistics of the leukemia patient.

Gene	type	point mutation	insertion	deletion	frame shift mutation	synonymous	noncoding	missense	total
ATPase8	Protein coding	1	0	0	0	1	0	0	1 (1.02%)
ATPase6	Protein coding	2	0	0	0	0	0	2	2 (2.04%)
COI	Protein coding	3	0	0	0	3	0	0	3 (3.06%)
COII	Protein coding	2	0	0	0	1	0	1	2 (2.04%)
COIII	Protein coding	2	0	0	0	2	0	0	2 (2.04%)
Cytb	Protein coding	5	0	0	9	3	0	2	14 (14.29%)
ND1	Protein coding	1	0	0	0	1	0	0	1 (1.02%)
ND2	Protein coding	1	0	0	0	1	0	0	1 (1.02%)
ND3	Protein coding	3	0	0	0	1	0	2	3 (3.06%)
ND4	Protein coding	3	0	0	0	3	0	0	3 (3.06%)
ND5	Protein coding	2	0	1	1	1	0	1	4 (4.08%)
ND6	Protein coding	17	0	0	15	7	2	8	32 (32.65%)
12S RNA	ribosomal RNA	2	0	0	0	0	0	0	2 (2.04%)
16S RNA	ribosomal RNA	1	0	1	0	0	0	0	2 (2.04%)
NC3	transfer RNA	0	0	2	0	0	2	0	2 (2.04%)
A	Transfer RNA	0	1	2	0	0	0	0	3 (3.06%)
N	Transfer RNA	1	0	1	0	0	0	0	2 (2.04%)
OL	Transfer RNA	0	0	1	0	0	1	0	1 (1.02%)
E	Transfer RNA	1	0	0	0	0	0	0	1 (1.02%)
D-Loop	No coding	11	5	1	0	0	17	0	17 (17.35%)
total		58	6	9	25	24	22	16	98 (100%)

## 3 Discussion

In this study, the complete genomic mutations of platelet mitochondria were analyzed for the first time in a benzene-induced leukemia patient. It was reported that the mutation frequencies of mitochondrial genes among the normal group were less than 5%.^[13]^ This study found that 3 mitochondrial genes, that are ND6, Cytb, and D-Loop, had mutation frequencies of more than 5% in platelets of the leukemia patient. In particular, ND6 gene, which is a protein-coding gene, had the highest mutation frequency of up to 32.65%. The Cytb is also a protein-coding gene, and the D-Loop region has a very significant transcriptional regulation function.

Studies have shown that altered nucleotides may affect the binding of mtDNA to transcription factors, resulting in abnormal mtDNA and protein complex formation, thus changing the normal expression of mtDNA gene.^[14]^ And some scholars found that mitochondrial mutation rate and the occurrence of cancer were positively correlated.^[15]^ Many experimental studies have investigated the association between mutations in mitochondrial genes and the risk of leukemia. The high rate of mutations was found in D-loop region and in the Cytb gene in pediatric leukemia patients.^[16]^ Significant increases of leukemia risk were observed for the ATP synthase subunit 6 gene and ND2 gene mutations of mtDNA. ^[17,18]^

High ND6 mutation frequency was first reported in leukemia in this study. ND6 gene encodes ND6 subunit, which is 1 of the 40 subunits of the NADH dehydrogenase (also known as complex I), in mammalian cells.^[19]^ NADH dehydrogenase activity was associated with polymorphism or mutations of ND6 gene.^[20]^ NADH dehydrogenase deficiency may cause maternally inherited diseases such as Leber hereditary optic neuropathy and mitochondrial encephalomyopathy with lactic acidosis and stroke-like episodes. ND6 missense mutation has been reported to contribute to tumor cell metastasis in mouse fibrosarcoma, lung carcinoma and colon cancer.^[21]^ The association of ND6 mutation with leukemia has never been reported before. It is possible that the ND6 mutation specifically links to benzene-induced leukemia. This case study is only a preliminary study. The study did not include drug sensitivity surveillance for the patient's response to imatinib treatment. Although there have been reports of mitochondrial gene mutations related to imatinib resistance in CML,^[22]^ the same mutation was not found in this case study.

Although the current gene sequencing technology has developed to the third generation, Sanger sequencing is still the gold standard for sequence identification. This study used Sanger sequencing technology to ensure the accuracy of sequencing results. In this study, nine primers were designed, and the primers were overlapped, so that the amplification efficiency of the mitochondrial hypervariable region was solved. The platelet mitochondrial genes were amplified by 99.8%. Platelets do not contain nuclei, and therefore, the mitochondrial genome is the only genetic material from platelets. Therefore, sequencing of mitochondrial genes in platelets is not affected by nucleus genome, which is critical for understanding their implication in the development of diseases.

In summary, the platelet mitochondrial genome was tested in a benzene-induced leukemia patient for the first time in this report. Many mutations were identified with PCR and confirmed by the Sanger sequencing. In future studies, the relation of the platelet mtDNA mutations with the clinical phenotype, treatment and outcomes of benzene-induced leukemia would need further investigation.

## Acknowledgments

The authors would like to extend our thanks to all participants of this study.

## Author contributions

**Data curation**: Diya Cai.

**Formal analysis**: Dianpeng Wang, Xiangli Yang, Peimao Li.

**Investigation**: Zhimin Zhang.

**Methodology**: Dianpeng Wang, Xiangli Yang.

**Project administration**: Dafeng Lin.

**Supervision**: Yanfang Zhang, Dafeng Lin.

**Writing – original draft**: Dianpeng Wang.

**Writing – review & editing**: Dafeng Lin

## Supplementary Material

Supplemental Digital Content

## Supplementary Material

Supplemental Digital Content
