# Supplementary material for: Genomic analysis of mutations in platelet mitochondria in a case of benzene-induced leukaemia: A case report
Source: Medicine (Baltimore). 2021 Jan 8;100(1):e24014. doi: 10.1097/MD.0000000000024014 (PMC7793417; doi:10.1097/MD.0000000000024014)
Supplement: Supplemental Digital Content [file medi-100-e24014-s001.docx]

**Supplementary Table 1. Primer sequences**

| primer | | sequence(5'->3') | start | end | product |  |
| --- | --- | --- | --- | --- | --- | --- |
| A | forward | GATCACAGGTCTATCACCCTATTA | 1 | 24 | 1949 | |
|  | reverse | CGGGTGTGCTCTTTTAGCTG | 1949 | 1930 |  |  |
| B | forward | TAAGACCCCCGAAACCAGAC | 1896 | 1915 | 1955 | |
|  | reverse | CCAAGGGTCATGATGGCAGG | 3850 | 3831 |  |  |
| C | forward | TCTCCACCCTTATCACAACACA | 3791 | 3812 | 1951 | |
|  | reverse | CGGCGGGAGAAGTAGATTGA | 5722 | 5741 |  |  |
| D | forward | CACTTAGTTAACAGCTAAGCACCC | 5685 | 5708 | 1983 | |
|  | reverse | GGGCGTGATCATGAAAGGTG | 7667 | 7648 |  |  |
| E | forward | CATGCAGCGCAAGTAGGTCT | 7592 | 7611 | 1996 | |
|  | reverse | TAGGGGATTTAGCGGGGTGA | 9587 | 9568 |  |  |
| F | forward | CCCGCTAAATCCCCTAGAAG | 9536 | 9555 | 1976 | |
|  | reverse | GGGGGTTGAGAATGAGTGTG | 11511 | 11491 |  |  |
| G | forward | CGGCTATGGTATAATACGCCTCA | 11476 | 11498 | 1988 | |
|  | reverse | CCAATGGTGAGGGAGGTTGA | 13463 | 13444 |  |  |
| H | forward | TATGTGCTCCGGGTCCATCA | 13356 | 13375 | 1327 | |
|  | reverse | GGTCGTGGTTGTAGTCCGTG | 14701 | 14682 |  |  |
| I | forward | GAGAAGGCTTAGAAGAAAAC | 14620 | 14601 | 1969 | |
|  | reverse | CATCGTGATGTCTTATTTAAGGGGA | 16569 | 16546 |  |  |
