# Supplementary material for: Genomic analysis of mutations in platelet mitochondria in a case of benzene-induced leukaemia: A case report
Source: Medicine (Baltimore). 2021 Jan 8;100(1):e24014. doi: 10.1097/MD.0000000000024014 (PMC7793417; doi:10.1097/MD.0000000000024014)
Supplement: Supplemental Digital Content [file medi-100-e24014-s002.docx]

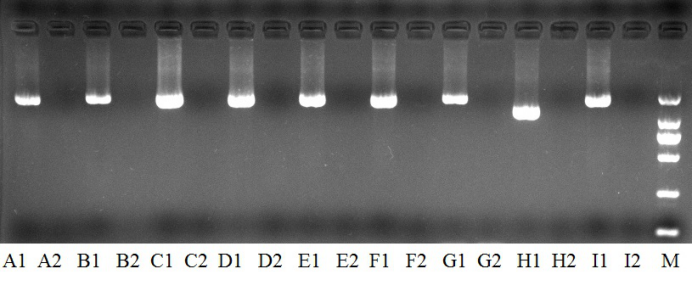
**Supplementary Figure 1** Platelet mitochondrion whole-gene fragmentation electrophoresis (A1 is a pair of primers to amplify a patient sample, A2 is A a pair of primers to amplify a negative control, and so on).
